# Grape berries biochemical shifts from vines under summer stress treated with kaolin and silicon mixtures

**DOI:** 10.3389/fpls.2025.1681593

**Published:** 2025-10-29

**Authors:** Sandra Pereira, Miguel Baltazar, Zélia Branco, Ana Monteiro, A. Sérgio Serrano, Adela Mena Morales, Rebeca Cruz, Susana Casal, Damián Balfagón, Renata Moura, José Moutinho-Pereira, Lia-Tânia Dinis

**Affiliations:** ^1^ Centre for the Research and Technology of Agro-Environmental and Biological Sciences (CITAB), University of Trás-os-Montes e Alto Douro (UTAD), Vila Real, Portugal; ^2^ Institute for Innovation, Capacity Building and Sustainability of Agri-Food Production (Inov4Agro), University of Trás-os-Montes e Alto Douro (UTAD), Vila Real, Portugal; ^3^ Regional Institute for Agri-Food and Forestry Research and Development of Castilla-La Mancha (IRIAF), Tomelloso, Spain; ^4^ LAQV-REQUIMTE, Department of Chemical Sciences, Faculty of Pharmacy, University of Porto, Porto, Portugal; ^5^ Department of Biology, Biochemistry and Natural Sciences, Universitat Jaume I, Castellón, Spain

**Keywords:** viticulture, kaolin, silicon, abiotic stress mitigation, secondary metabolites, grape berry quality, foliar application, cuticular waxes

## Abstract

**Introduction:**

Climate change is intensifying abiotic stress in viticulture, with higher temperatures, water deficits, and increased solar radiation directly affecting grape berry development, biochemical balance, and overall fruit quality. These challenges compromise the delicate balance of sugars, acids, phenolic compounds, and aromatic profiles that define the sensory attributes and typicity of the resulting wine. Innovative practices are therefore needed to sustain fruit quality and composition under increasingly variable and extreme climatic conditions. Silicon (Si) and kaolin (Kl) have already shown highly positive effects in mitigating the impacts of climate change on grapevines.

**Methods:**

Building on this, the aim of this study was to test their combined foliar application to simultaneously alleviate drought and heat stress and enhance berry quality. An integrated assessment was conducted over two growing seasons in a commercial vineyard (Vitis vinifera L. cv. Touriga Franca) located at Quinta de Ventozelo (Douro Demarcated Region, Portugal) to evaluate the effects of combined Kl (2%) and Si foliar sprays at different concentrations (2–8%). The study included analyses of fruit biochemical composition, must quality, cuticular wax profile, histological traits, carbon isotope discrimination (d^13^C), hormonal balance, and yield parameters.

**Results and discussion:**

Results showed that Si and Kl treatments modulated secondary metabolite accumulation (phenols, flavonoids, anthocyanins, and tannins) in a season- and stage-dependent manner, with significant increases under milder environmental conditions, particularly in seasons with lower heat and drought stress. This suggests that these products can act as elicitors or stress mitigators, depending on the environmental context. Treated vines maintained higher organic acid levels and lower probable alcohol content, indicating an improved sugar–acid balance. Moreover, the treatments influenced the cuticular wax composition, enhancing triterpenoid content and increasing cuticle thickness and epidermal cell size, which, together with enriched d^13^C values, support improved water-use efficiency. The hormonal profiles confirmed the role of Si and Kl in fine-tuning stress and growth signals, contributing to better fruit robustness.

**Conclusions:**

These findings demonstrate that the combined foliar application of kaolin and silicon is a promising tool to protect grape berry quality by modulating biochemical composition, cuticular wax profile, histological traits, isotopic signature, and hormonal balance, helping maintain fruit integrity and quality, and compositional stability under the ongoing challenges of climate change.

## Graphical Abstract

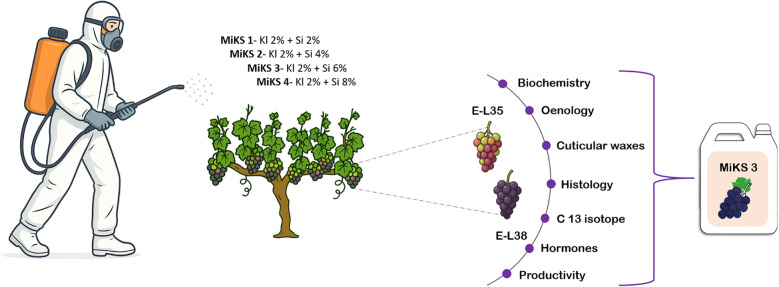

## Introduction

Climate change is increasingly altering grapevine development, with serious consequences for grape yield and wine quality. Temperature and precipitation, two critical climatic factors, strongly influence the onset of ripening, affecting fruit development and quality (Luzio et al., 2021; Bernardo et al., 2022). These effects are particularly pronounced in regions with Mediterranean climates, which are considered climate change hotspots (Conde et al., 2007; Kuhn et al., 2014; Leolini et al., 2020; Santos et al., 2020). Characterized by hot, dry summers with limited precipitation, these areas usually face intensified drought stress, impairing grapevine physiology, and reducing both berry quality and vineyard productivity. Furthermore, global warming is leading to earlier grape maturation, which has been documented across many of these viticultural regions (Duchêne and Schneider, 2005; Wolfe et al., 2005; Etien et al., 2009; Duchêne et al., 2010; Urhausen et al., 2011; Webb et al., 2011; Morales-Castilla et al., 2020). This shift is largely driven by summer stress, which stimulates the production of abscisic acid (ABA), a key hormone in the ripening process (Chaves et al., 2009). Increased temperatures can also cause sugar accumulation to outpace the development of phenolic and aromatic compounds, resulting in a mismatch between sugar accumulation and phenolic synthesis (Kliewer and Torres, 1972; Van Leeuwen and Seguin, 2006; Goode, 2012; Sadras and Moran, 2012). This imbalance may prevent the full development of anthocyanin and tannin, whose accumulation is favored by cooler temperatures, while higher temperatures are known to decrease their synthesis or promote their degradation. Additionally, high temperatures accelerate the degradation of malic acid, especially during warm nights, further disrupting the balance between sugars, acids, and aromas (Gatti et al., 2014; Leolini et al., 2019).

Grape ripening is tightly regulated by hormonal dynamics, which orchestrate the synthesis of secondary metabolites essential for redox homeostasis and wine quality (Kuhn et al., 2014; Soto et al., 2015). Among these hormones, ABA plays a pivotal role, particularly under environmental stressors such as heat, intense light, UV-B radiation, and water deficit (Fortes et al., 2015; Dinis et al., 2018a). While ABA accumulation is a well-known marker of ripening onset, recent findings highlight its integration within a complex hormonal signaling network, involving cross-talk with other growth regulators (Kuhn et al., 2014; Pérez-Llorca et al., 2019). Under abiotic stress, the levels of indole-3-acetic acid (IAA) tend to decline, limiting cell expansion and berry growth but facilitating the ripening process, as elevated IAA is known to delay maturation (Davies, 2010; Böttcher et al., 2010). Conversely, salicylic acid (SA) generally increases in response to stress, activating defense mechanisms. However, its antagonistic interaction with ABA can hinder ripening and pigment accumulation, potentially compromising berry quality (Miura and Tada, 2014; Li et al., 2025). Jasmonic acid (JA) also tends to rise under stress conditions such as heat and drought, promoting the accumulation of phenolic compounds and anthocyanins, thereby enhancing fruit quality (Fortes et al., 2015; Wasternack and Strnad, 2019). Prolonged or excessive JA signaling may divert resources from growth to defense, resulting in yield reduction (Munné-Bosch and Muller, 2013; Campos et al., 2016). Altogether, stress-induced hormonal adjustments influence critical traits such as anthocyanin biosynthesis, berry size, sugar accumulation, and the timing of ripening (Niculcea et al., 2013; Parada et al., 2017). Besides hormonal regulation, berry structural features also contribute to stress resilience. For instance, cuticle thickness, cuticular wax composition, and epidermal tissue characteristics can influence water loss, oxidative stress protection, and fruit firmness, determining the grape’s ability to withstand heat and drought stress (Lara et al., 2014; Zhang et al., 2021). As climate change is increasingly challenging viticulture, there is an urgent need for adaptive strategies to preserve grape quality and ensure vineyard sustainability under climate pressure. Among these, foliar applications of kaolin (Kl) and silicon (Si) have emerged as promising tools to reduce thermal and water stress, support physiological function, and maintain berry quality. Kl is a natural occurring inert clay mineral rich in kaolinite, widely used in agriculture for its eco-friendly properties and versatility. When applied as a white foliar coating, it increases solar reflectance, which lowers leaf temperature, minimizes photoinhibition, and protects against thermal damage (Glenn et al., 2010; Dinis et al., 2016b; Luciani et al., 2020). In grapevines, these effects translate into improved photosynthetic performance and water retention, contributing to better berry composition under abiotic stress (Shellie and Glenn, 2008; Dinis et al., 2018b). Additionally, Kl forms a protective barrier that limits pest and pathogen attacks (Brito et al., 2019; Linder et al., 2020; Rashad et al., 2023). Its benefits in enhancing fruit quality have also been demonstrated in various crops such as apples, pomegranates, tomatoes, and olives (Saour and Makee, 2003; Melgarejo et al., 2004; Cantore et al., 2009).

Although not considered an essential nutrient, Si is increasingly recognized as a beneficial biostimulant that enhances plant tolerance to abiotic stress (Pereira et al., 2024). Absorbed by plants in the form of orthosilicic acid, Si accumulates in tissues where it reinforces cell walls and contributes to both structural integrity and biochemical defense mechanisms (Ma and Yamagi, 2006; Coskun et al., 2018). Its roles include improving nutrient uptake, enhancing photosynthetic capacity, stabilizing cellular membranes, and promoting osmotic adjustment. Si also activates antioxidant enzymes and facilitates the scavenging of reactive oxygen species (ROS), especially under drought, salinity, and biotic stress. Moreover, it interacts with key phytohormones such as ABA, strengthening the plant’s stress signaling pathways (Debona et al., 2017; Rastogi et al., 2021). In grapevines, Si application has been associated with improved water-use efficiency, better-regulated stomatal conductance, reinforced cell walls (Dinis et al., 2024a), delayed senescence, and increased resilience to environmental challenges (Gunes et al., 2008; Ma and Yamaji, 2015; Rizwan et al., 2019).

Based on the known stress-mitigation effects of Si and Kl, we hypothesize that their combined foliar application can modulate key metabolic pathways and enhance berry quality, while also promoting physiological resilience under variable stress conditions. In this sense, the objective of this study was to evaluate the effects of the combined application of Kl (2%) and Si (2-8%) on the biochemical composition, cuticular wax content, fruit histology, carbon isotope composition (δ¹³C), hormonal profile, and yield of grape berries under summer stress conditions.

## Material and methods

### Field trail and experimental design

The field trial was carried out in a commercial vineyard (Quinta de Ventozelo, 41°18.954′ N, 8°38.940′ W), located in Ervedosa do Douro, within the Cima Corgo sub-region of the Douro Demarcated Region. This area features a moderate Mediterranean climate, characterized by hot, dry summers and mild, wet winters, making it particularly suitable for producing high-quality Port and Douro wines. The vine training system was single Royat cordon and the vineyard was irrigated. The experiment was performed in two consecutive growing seasons (2023 and 2024) in a vineyard planted in 2014 with *Vitis vinifera* L. cv. Touriga Franca grafted onto 1103P rootstock. In 2023, four treatments were evaluated: an untreated control and three foliar formulations combining kaolin (Kl) with increasing silicon (Si) concentrations: MiKS 1- 2% Kl + 2% Si, MiKS 2- 2% Kl + 4% Si, and MiKS 3- 2% Kl + 6% Si. The experimental layout followed a randomized block design with three replicates per treatment (12 rows in total), each consisting of 15 vines. Based on the 2023 outcomes, the 2024 trial excluded MiKS1 and introduced a new formulation, MiKS 4 (2% Kl + 8% Si), while maintaining the remaining treatments. Foliar applications were performed twice per season, at two-week intervals, in end-June (E-L32) and early July (E-L34), spraying the whole canopy.

### Bunch number, yield, and bunch mass

At E-L38, the number of bunches per vine and the yield (kg vine^−^¹) were assessed in fifteen treated vines per treatment. Additionally, the average bunch weight was also determined. The decision on harvest date was made by Quinta de Ventozelo, based on their own routine monitoring of grape ripening.

### Chemical analysis of grape juice

For the chemical analysis of grape juice, three replicates of 100 fresh berries (one replicate per plot) at two phenological stages (E-L35 – *veraison* and E-L38 – harvest) were collected. The probable alcohol content was analyzed according to OIV methodologies (OIV, 2003). Total acidity was quantified using Fourier Transform Infrared Spectroscopy (FTIR) (OIV, 2010), and pH was measured with a pH meter (3310 Jenway, UK). *L*-malic and tartaric acid concentrations were determined using an enzymatic method with an automated clinical chemistry analyzer (Miura One, TDI, Spain) following the procedure of Escribano-Viana et al. (2019).

### Fruit metabolites quantification

For each treatment, three fruit samples were collected at two phenological stages (E-L35 – *veraison* and E-L38 – harvest), each comprising approximately 15 berries, with one sample obtained from each experimental replicate (15 vines per row x 3 replicates, totaling 45 vines per treatment). Immediately after collection, samples were flash-frozen in liquid nitrogen in the field. In the laboratory, berries were lyophilized, and all analytical results are reported on a dry weight basis. Following lyophilization, samples were ground into a fine powder under liquid nitrogen.

#### Quantification of phenolic and flavonoid compounds

To determine phenolic and flavonoid compounds, a methanolic extract at a concentration of 4 mg of lyophilized sample per ml was prepared and used for the following quantifications.

Total phenols were quantified using the Folin–Ciocalteu method, with absorbance measured at 725 nm (Rodrigues et al., 2015). Results were expressed in milligrams of gallic acid equivalents per g of dry weight (mg GAE g^−^¹ DW).

Total flavonoid content in the extracts was quantified using the aluminum chloride (AlCl_3_) complex method, with absorbance measured at 510 nm, as described by Rodrigues et al. (2015). Results were expressed as milligrams of catechin equivalents per gram of dry weight (mg CAE g^−^¹ DW).

#### Anthocyanins and tannins quantification

The total anthocyanin content was quantified using the differential pH method, which is based on its pH-dependent color changes (Lee et al., 2005). Absorbance readings were taken at specific wavelengths under different pH conditions, allowing for the calculation of the total anthocyanin content. Results were expressed as milligrams of cyanidin-3-glucoside equivalents per gram of dry weight (mg cyd-3-glu g^−^¹ DW).

The content of condensed tannins was determined using the vanillin-HCl assay (Dambergs et al., 2012), which is based on their reaction with vanillin in the presence of hydrochloric acid. The absorbance was measured at 500 nm, and the concentration of condensed tannins was calculated using a standard curve prepared with catechin. The results were expressed as milligrams of catechin equivalents per gram of dry weight (mg CAE g^−^¹ DW).

### Grapevine fruit cuticular waxes

#### Extraction and chemical characterization

The total amount of cuticular waxes was quantified following the method described by Riederer and Schreiber (1995). Fresh berries were collected and stored at 4°C until analysis. Samples were grouped into five sets, each containing five fruits. Cuticular waxes from each fruit group were extracted by immersing the samples in 50 mL of chloroform in glass tubes for 2 minutes. After evaporation of the chloroform, the wax residue was weighed, and quantities were expressed relative to the total fruit surface area and total mass.

For chemical analyses, an internal standard of tetracosane was added to the dried composite wax extract, which were then re-dissolved in chloroform. An aliquot (100 µL) was evaporated to dryness under a nitrogen stream and derivatized with a 1:1 (v/v) mixture of bis-trimethylsilyltrifluoroacetamide (BSTFA, CF_3_C[=NSi(CH_3_)_3_]OSi(CH_3_)_3_) and pyridine at 70°C for 30 minutes, converting hydroxyl groups to their corresponding trimethylsilyl (TMS) derivatives.

Chromatographic separation was performed on a gas chromatograph (Agilent 7890A, USA) equipped with an HP5-MS column (30 m × 0.25 mm I.D. × 0.25 μm film thickness, Supelco, USA) using helium as the carrier gas, with a temperature gradient from 80°C to 325°C and injection (1 µL) at 280°C in pulsed splitless mode. System performance was regularly verified using an Alkane Standard Mixture (C_10_; C_20_ - C_40,_ all even, 50 mg L^-1^, Supelco 94234, Sigma-Aldrich, USA).

Compound detection was achieved with a 5977B MSD mass detector using electron ionization at 70 eV in full scan mode (m/z 50–650). Identification relied on available standards and computer matching against the NIST Mass Spectral Library 11.

For quantification, detection was switched to a flame ionization detector (FID, Agilent 7890A, USA) set at 280°C. Quantification (µg g^−^¹ FW) was based on the response of an internal standard of known concentration. When available, individual standards were used to determine response factors, which were applied to structurally similar compounds with close retention times. Results are expressed as both estimated concentrations and relative peak areas.

Multiple wax classes were identified on the cuticular surfaces. Unknown compounds were tentatively identified using the NIST library’s Match Factor and Reversed Match Factor, where higher values indicate more reliable matches. Procedural blanks were analyzed to detect interferences, such as bibenzyl. ELSS screening further identified contaminants including bis(2-ethylhexyl) phthalate, cis-13-docosenamide, and tris(2,4-di-tert-butylphenyl) phosphate, which were excluded from total area calculations as they likely originated from the extraction process. To detect potential contaminants from packaging materials and tubing, an extractable and leachable screening standard (ELSS, TraceCERT^®^, Sigma-Aldrich, USA) was used to monitor volatile and semi-volatile compounds.

Octanol/water partition coefficients (K_OW_) for individual chemicals identified in the cuticular wax extracts were calculated using the EPI Suite™ 6.3 software (EPA, USA).

### Histological traits

Anatomical tissue measurements were conducted on six berries per treatment, with two fruits collected from each of the three replicates, only at E-L38 phenological stage (harvest) of 2024. After fixation in formalin aceto-alcohol for 48 hours, the samples underwent dehydration, clearing, and paraffin embedding. Transverse sections (4μm thick) were then prepared using a rotary microtome (Leica RM 2135, Germany). These sections were mounted on slides and stained with 0.1% toluidine blue. Tissue thickness was measured using an inverted optical microscope (Olympus IX51, Olympus Corporation, Tokyo, Japan) and analyzed with Digimizer image analysis software (MedCalc Software, Ostend, Belgium).

### δ¹³C isotopic quantification

δ¹³C isotopic quantification was conducted exclusively on berries (1 sample of 15 berries per plot x 3 replicates) collected at E-L38 phenological stage (harvest) of each growing season.

The carbon isotope quantification was measured by online analysis using a ThermoQuest Flash 1112 Elemental Analyzer equipped with an autosampler and coupled to a Delta-Plus IRMS (ThermoQuest, Bremen, Germany) through a ConFlo III interface (ThermoQuest). One microliter of must was placed in a tin capsule and sealed. All the carbon in the sample was oxidized to CO_2_ by the reactors of the elemental analyzer. The analyzer passed the gas through a gas chromatography (GC) column to separate the CO_2_ from other gases and then brought the CO_2_ into the mass spectrometer by a helium flow. The carbon isotope composition was expressed as (Eqn 1):


$${\text{δ}}^{13}\text{C }=[({\text{R}}_{\text{s}}/{\text{R}}_{\text{std}})-1]1000$$

where R_s_ is the ^13^C/^12^C ratio of the sample and R_std_ is the international reference standard Vienna Pee Dee Belemnite (VPDB).

### Phytohormone composition

The contents of abscisic acid (ABA), indole-3-acetic acid (IAA), jasmonic acid (JA), and salicylic acid (SA) were determined, in E-L32 – beginning of bunch closure, E-L35 – *veraison* and E-L38 – harvest penological stages of 2024 (three samples of 15 berries), using high-performance liquid chromatography coupled with a triple quadrupole mass spectrometer (Micromass^®^, Manchester, UK) via an orthogonal Z-spray electrospray ion source (Durgbanshi et al., 2005). Approximately 20 mg of lyophilized fruits were extracted in 2.0 mL of distilled water using a mill ball apparatus (MillMix20, Domel, Železniki, Slovenia). [²H_6_]-ABA, [²H_5_]-IAA, dihydrojasmonic acid (DHJA) and [¹³C_6_]- SA (Sigma-Aldrich, USA) were used as internal standards.

After centrifugation at 10,000 ×*g*, the supernatants were collected, and the pH was adjusted to 2.8–3.2 with 30% acetic acid. Extracts were partitioned twice with diethyl ether, and the resulting supernatants were evaporated under vacuum using a centrifuge concentrator (Speed Vac, Jouan, Saint Herblain Cedex, France) at room temperature. The dry residue was resuspended in 500 μL of a 9:1 water:methanol solution, filtered through 0.22 μm PTFE filters, and directly injected into an ultra-performance liquid chromatography system (Waters™ Acquity SDS, Waters Corporation, Milford, MA, USA) interfaced with a TQD triple quadrupole mass spectrometer (Micromass^®^ Ltd., Manchester, UK).

Chromatographic separation was performed on a reversed-phase C18 column (Gravity, 50 × 2.1 mm, 1.8 μm particle size; Macherey-Nagel GmbH, Germany) using a methanol:water gradient supplemented with 0.1% acetic acid at a flow rate of 300 μL min^−^¹. The aqueous phase was maintained at 90% for the first 2 minutes, decreased to 10% over 6 minutes, then increased back to 90% by 7 minutes, and held constant until the end of the 8-minute run.

Mass spectrometry was conducted in multiple reaction monitoring mode using nitrogen as the drying and nebulizer gas (cone gas flow: 250 L h^−^¹; desolvation flow: 1200 L h^−^¹) and argon as the collision gas. Cone voltage and collision energies were set according to Durgbanshi et al. (2005), with minor modifications. Data processing was carried out using MassLynx™ v4.1 software, and phytohormone concentrations were determined by interpolating the response ratios of the phytohormones to their internal standards against a calibration curve prepared with commercial ABA, IAA, JA, and SA standards.

### Statistical analyses

Data analysis was performed using the SPSS 20.0 software (SPSS Software, Chicago, IL, USA). After testing for analysis of variance (ANOVA) assumptions, statistical differences among treatments within each developmental stage and year were evaluated by one-way factorial ANOVA, followed by the *post hoc* Tukey test. Different letters represent significant differences (*P* < 0.05) among the applied formulations.

## Results and discussion

The application of combined Si and Kl formulations significantly influenced multiple physiological, biochemical, and anatomical characteristics of grapevines (Pereira et al., 2025), with variable responses across the two growing seasons and phenological stages. These findings underline the complex and context-dependent interactions between foliar treatments and plant metabolism leading to changes in fruit metabolism.

### Climatic conditions

**Figure 1:**
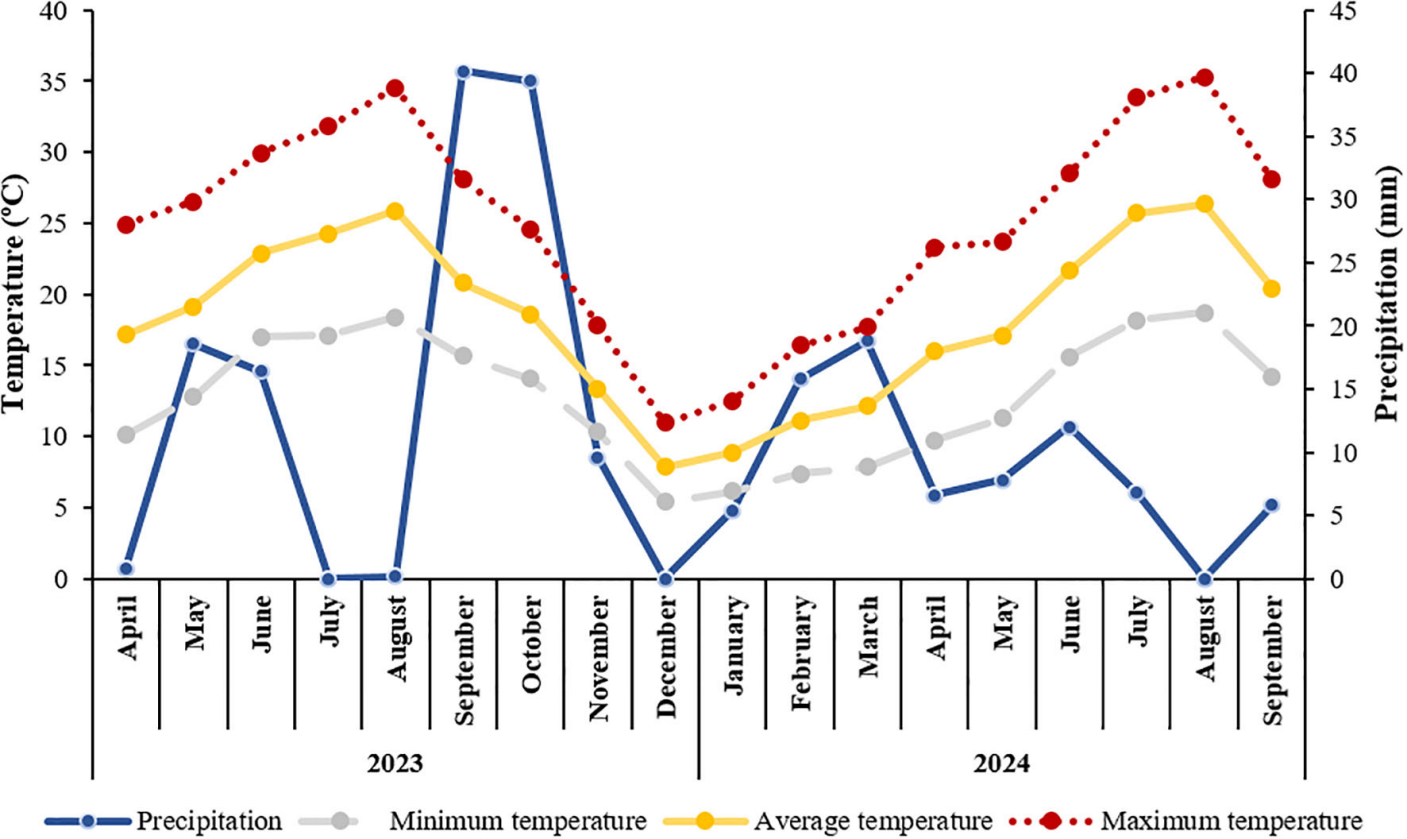
Monthly precipitation (mm) and mean air temperature (minimum, average and maximum) (ºC) of the experimental site, collected by an on-site weather station, from April 2023 to September 2024.

Meteorological data, comprising precipitation and minimum, average, and maximum temperatures, were recorded throughout the experimental period by an on-site weather station and are summarized in **Figure 1**.

In 2023, average air temperature increased progressively from around 17°C in April to a peak of 26°C in August, before declining to 8°C by December. Minimum monthly temperatures ranged from 5.4°C (December) to 18°C (August), while maximum values reached up to 35°C in August. The summer was notably dry. The highest rainfall occurred in September and October, but the July–August period was marked by a complete absence of rainfall, coinciding with peak temperatures. These conditions suggest elevated drought stress, potentially affecting vine physiology and irrigation demand.

In contrast, 2024 presented cooler initial conditions, with an average temperature of 9°C in January, rising to 26°C in August, and declining to 20°C in September. Minimum monthly temperatures ranged from 6°C (January) to 19°C (August), while maximum temperatures varied from 13°C (January) to 35°C (August), before dropping to 28°C in September. Precipitation was lower overall compared to 2023, with the highest monthly total (around 19 mm) recorded in March and no rainfall in August.

According to the geoclimatic classification proposed by Tonietto and Carbonneau (2004), the experimental site in the Douro Demarcated Region corresponded to a very warm and dry viticultural climate in 2023 (HI = 2895.5; DI = –30) and to an extremely warm and dry climate in 2024 (HI = 3855.8; DI = –54.9), based on local climatic data.

### The impact of formulations in bunch number, yield, and bunch mass

**Figure 2:**
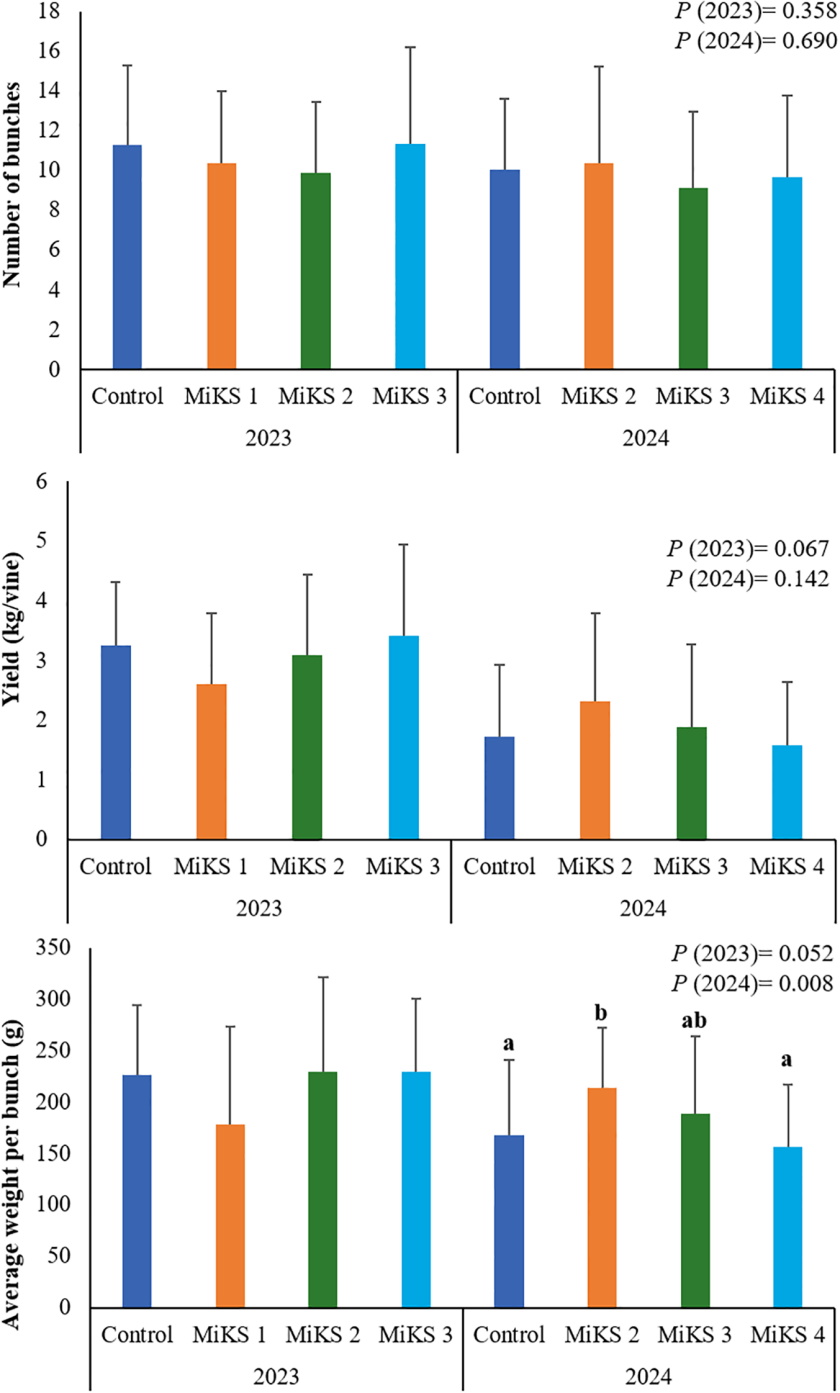
Number of clusters, yield and average weight per bunch of vines with different foliar applications: untreated plants (Control), plants treated with Kl 2% and Si 2% (MiKS 1), plants treated with Kl 2% and Si 4% (MiKS 2), plants treated with Kl 2% and Si 6% (MiKS 3), and plants treated with Kl 2% and Si 8% (MiKS 4) (N=15). Results are expressed as mean ± standard deviation. Different letters indicate significant differences (*P* < 0.05) and the absence of letters indicates no statistically significant differences (*P* > 0.05) between treatments, within the same year, according to Tukey’s test.

Regarding yield (**Figure 2**), significant differences were observed only in 2024 for the parameter average weight per bunch, with MiKS 2 showing a significantly higher value than both the control plants and those treated with MiKS 4, representing increases of 27.0% and 36.7%, respectively. An increase in cluster weight following foliar application of Si has also been reported by Schabl et al. (2020), supporting the potential of Si-based formulations to enhance yield components under specific conditions. The absence of a similar increase for MiKS 4-treated plants, despite its positive effects on physiological (Pereira et al., 2025), biochemical, and anatomical traits, may indicate a trade-off between stress resilience and biomass allocation to fruit under certain environmental scenarios.

### Impacts of Kl and Si applications on the chemical composition of berries

**Table 1** presents the chemical composition of grape juice from plants treated with different Kl and Si formulations, evaluated at the E-L35 and E-L38 phenological stages in both 2023 and 2024. During E-L35 in 2023, no significant differences were observed in probable alcohol, pH, or malic and tartaric acid contents across treatments. However, total acidity showed significant variation, with MiKS 3-treated plants presenting the highest values, corresponding to a 10.6% increase compared to MiKS 1. In this study, although no statistically significant differences were observed, a slightly reduction was noted for tartaric acid in MiKS 1, and malic acid in MiKS 2-treated plants. At E-L38 in 2023, significant differences between treatments were observed only for tartaric acid content, with MiKS 2-treated plants exhibiting significantly higher levels than those treated with MiKS 3, representing a 21.4% increase. However, this increase did not translate into differences in total acidity. During E-L35 in 2024, untreated vines and those treated with the highest Si concentrations (MiKS 3 and MiKS 4) showed the highest probable alcohol content, exceeding the levels observed in MiKS 2-treated vines by 16.9%, 25.4%, and 17.2%, respectively. Malic acid followed a trend similar to total acidity, with MiKS 2 and MiKS 4 displaying the highest concentrations. Additionally, MiKS 4 also presented the highest tartaric acid content. At E-L38 in 2024, untreated plants and those receiving the lowest Si dose (MiKS 2) exhibited the highest probable alcohol content, significantly surpassing the levels observed in plants treated with higher Si doses (MiKS 3 and MiKS 4). Consequently, plants treated with MiKS 3 and MiKS 4 displayed increased tartaric and malic acid levels. Specifically, malic acid concentrations were higher compared to MiKS 2 plants (21.3 and 13.3% increase for MiKS 3 and MiKS 4, respectively) while tartaric acid levels were significantly greater than those in untreated and MiKS 2-treated vines. The observed increases in both tartaric and malic acids, alongside decreases in probable alcohol content in MiKS 3- and MiKS 4-treated plants during E-L38 in 2024 suggest a shift in carbon allocation and sugar–acid balance. This pattern may be associated with reduced sugar accumulation in the berries, potentially linked to enhanced metabolic activity or shifts in carbon partitioning during grape berry ripening (Conde et al., 2007), although no direct measurements of source–sink relationships were performed in this work. These changes are particularly relevant in the current context of climate change, where rising temperatures tend to accelerate sugar accumulation and reduce organic acids in grapes, often leading to wines with excessively high alcohol levels and poor freshness. In fact, higher organic acid content is associated with improved sensory profiles, such as enhanced crispness and longevity, while lower alcohol wines are increasingly demanded by health-conscious consumers and required to meet market and regulatory expectations (Van Leeuwen and Darriet, 2016; Poni et al., 2018; Roy and Ash, 2022). An increase in tartaric acid content following Si application has been reported by several authors, further supporting the positive role of Si in organic acid retention in grape berries (Losada et al., 2022; Sut et al., 2022; Dinis et al., 2024a). Additionally, a reduction in°Brix, and consequently in probable alcohol content, has also been reported by Collins et al. (2024). Likewise, Dinis et al. (2024b) observed significant increases in tartaric acid content along with reductions in wine alcohol levels following the application of Kl. The reduction in probable alcohol content can be attributed to the shading effect provided by Kl, which lowers berry temperature, reduces water loss, and may delay the ripening process (Coniberti et al., 2013). Furthermore, the higher tartaric acid levels may result from decreased acid degradation due to the healthier canopy protecting the berries from excessive sun exposure, as well as the reflective properties of the Kl coating itself (Dinis et al., 2020).

**Table 1:** Oenological parameters (probable alcohol, total acidity, pH and organic acids content) evaluated in berries from vines with different foliar applications: with different applications: untreated plants (Control), plants treated with Kl 2% and Si 2% (MiKS 1), plants treated with Kl 2% and Si 4% (MiKS 2), plants treated with Kl 2% and Si 6% (MiKS 3), and plants treated with Kl 2% and Si 8% (MiKS 4) (N=3). Results are expressed as mean ± standard deviation. Different letters indicate significant differences (P < 0.05) and the absence of letters indicates no statistically significant differences (P > 0.05) between treatments, within the same year and phenological stage, according to Tukey’s test.

Year	Phenological stage	Treatment	Phenols (mg g^-1^)	Flavonoids (mg g^-1^)	Anthocyanins (mg g^-1^)	Tannins (mg g^-1^)
2023	E-L35	Control	47.7 ± 0.564 b	38.1 ± 3.56 b	2.07 ± 0.087 b	33.5 ± 1.92 c
		MiKS 1 (Kl_2% + Si_2%)	30.5 ± 2.01 a	23.6 ± 2.18 a	2.16 ± 0.137 b	25.3 ± 1.50 b
		MiKS 2 (Kl_2% + Si_4%)	32.9 ± 0.361 a	25.5 ± 1.14 a	1.81 ± 0.063 a	20.5 ± 0.965 a
		MiKS 3 (Kl_2% + Si_6%)	47.6 ± 1.60 b	46.8 ± 2.39 c	1.86 ± 0.068 a	41.2 ± 0.849 d
		*P* value	<0.001	<0.001	<0.001	<0.001
	E-L38	Control	32.6 ± 1.68 ab	43.3 ± 1.71 d	2.90 ± 0.063 c	25.1 ± 2.36 c
		MiKS 1 (Kl_2% + Si_2%)	28.8 ± 1.25 a	15.9 ± 1.30 a	3.13 ± 0.169 d	16.6 ± 1.14 a
		MiKS 2 (Kl_2% + Si_4%)	31.1 ± 0.974 ab	19.7 ± 0.591 b	2.67 ± 0.069 b	17.5 ± 1.55 a
		MiKS 3 (Kl_2% + Si_6%)	33.6 ± 4.36 b	22.8 ± 1.60 c	1.82 ± 0.017 a	21.0 ± 1.70 b
		*P* value	0.032	<0.001	<0.001	<0.001
2024	E-L35	Control	27.9 ± 0.218 c	29.9 ± 2.97 b	0.647 ± 0.063 a	12.3 ± 0.488 a
		MiKS 2 (Kl_2% + Si_4%)	26.2 ± 0.491 a	24.6 ± 1.92 a	1.24 ± 0.067 b	16.6 ± 1.99 b
		MiKS 3 (Kl_2% + Si_6%)	27.7 ± 0.569 bc	24.9 ± 2.33 a	2.33 ± 0.100 d	17.9 ± 0.686 b
		MiKS 4 (Kl_2% + Si_8%)	26.9 ± 0.568 ab	24.6 ± 1.00 a	1.87 ± 0.119 c	18.3 ± 1.75 b
		*P* value	<0.001	0.002	<0.001	<0.001
	E-L38	Control	20.0 ± 0.608 a	11.4 ± 1.10 a	5.68 ± 0.606 a	7.07 ± 1.37 a
		MiKS 2 (Kl_2% + Si_4%)	23.6 ± 0.515 b	15.4 ± 0.743 b	5.41 ± 0.224 ab	11.4 ± 1.71 b
		MiKS 3 (Kl_2% + Si_6%)	26.2 ± 0.521 c	14.2 ± 0.595 b	7.27 ± 0.910 bc	9.90 ± 1.32 b
		MiKS 4 (Kl_2% + Si_8%)	26.0 ± 0.481 c	14.3 ± 0.506 b	8.73 ± 0.393 c	9.83 ± 0.822 b
		*P* value	<0.001	<0.001	<0.001	0.001

E-L35, veraison; E-L38, maturation; Kl, kaolin; Si, silicon; MiKS, combined formulation with Kl and Si.

### Formulations effects on secondary metabolites of fruits

Secondary metabolites such as phenolics, flavonoids, anthocyanins, and tannins play a crucial role in grape berry chemical composition, contributing to antioxidant capacity, color, and stress resilience (Conde et al., 2016; Losada et al., 2022). The modulation of secondary metabolites such as total phenols, flavonoids, 2, and tannins by Si and Kl combined application indicates a notable influence on berry biochemical quality. **Table 2** summarizes the content of secondary metabolites in grapevine berries treated with different MiKS formulations during E-L35 and E-L38 phenological stages in both 2023 and 2024.

**Table 2:** Secondary metabolites (phenolics, flavonoids, anthocyanins and tannins) evaluated in berries from vines with different foliar applications: untreated plants (Control), plants treated with Kl 2% and Si 2% (MiKS 1), plants treated with Kl 2% and Si 4% (MiKS 2), plants treated with Kl 2% and Si 6% (MiKS 3), and plants treated with Kl 2% and Si 8% (MiKS 4) (N=9). Results are expressed as mean ± standard deviation. Different letters indicate significant differences (P < 0.05) and the absence of letters indicates no statistically significant differences (P > 0.05) between treatments, within the same year and phenological stage, according to Tukey’s test.

Year	Phenological stage	Treatment	Probable alcohol (% vol)	Total acidity (g L^-1^ tartaric acid)	pH	Malic acid (g L^-1^)	Tartaric acid (g L^-1^)
2023	E-L35	Control	7.28 ± 0.685	10.6 ± 2.56 bc	3.01 ± 0.140	3.77 ± 0.920	5.55 ± 1.58
		MiKS 1 (Kl_2% + Si_2%)	7.34 ± 0.250	9.88 ± 0.064 a	3.05 ± 0.050	3.42 ± 0.325	5.27 ± 0.338
		MiKS 2 (Kl_2% + Si_4%)	7.17 ± 0.514	10.5 ± 1.36 ab	3.03 ± 0.069	3.12 ± 0.342	6.23 ± 1.00
		MiKS 3 (Kl_2% + Si_6%)	7.05 ± 0.507	10.9 ± 1.97 c	2.98 ± 0.125	3.47 ± 0.774	6.27 ± 1.05
		*P* value	0.902	0.001	0.869	0.073	0.083
	E-L38	Control	12.8 ± 0.397	3.78 ± 0.157	3.84 ± 0.085	1.35 ± 0.531	1.66 ± 0.562 ab
		MiKS 1 (Kl_2% + Si_2%)	12.6 ± 0.025	3.75 ± 0.150	3.78 ± 0.040	1.39 ± 0.355	1.58 ± 0.510 ab
		MiKS 2 (Kl_2% + Si_4%)	12.3 ± 0.284	3.65 ± 0.229	3.77 ± 0.044	1.06 ± 0.323	1.87 ± 0.615 b
		MiKS 3 (Kl_2% + Si_6%)	12.0 ± 0.927	3.62 ± 0.312	3.76 ± 0.100	1.32 ± 0.165	1.54 ± 0.177 a
		*P* value	0.419	0.790	0.567	0.361	0.023
2024	E-L35	Control	6.31 ± 0.351 b	12.5 ± 0.110 a	2.85 ± 0.029	4.39 ± 0.310 a	6.27 ± 0.025 b
		MiKS 2 (Kl_2% + Si_4%)	5.40 ± 0.050 a	15.7 ± 0.060 c	2.84 ± 0.068	6.84 ± 0.379 b	5.81 ± 0.040 a
		MiKS 3 (Kl_2% + Si_6%)	6.77 ± 0.233 b	13.5 ± 0.340 b	2.89 ± 0.067	4.62 ± 0.315 a	5.66 ± 0.110 a
		MiKS 4 (Kl_2% + Si_8%)	6.33 ± 0.060 b	15.0 ± 0.415 c	2.87 ± 0.015	6.99 ± 0.870 b	7.02 ± 0.030 c
		*P* value	<0.001	<0.001	0.680	<0.001	<0.001
	E-L38	Control	12.9 ± 0.070 b	4.18 ± 0.117	3.83 ± 0.025	1.71 ± 0.051 b	1.76 ± 0.085 a
		MiKS 2 (Kl_2% + Si_4%)	12.7 ± 0.315 b	4.03 ± 0.155	3.71 ± 0.117	1.50 ± 0.064 a	1.77 ± 0.147 a
		MiKS 3 (Kl_2% + Si_6%)	12.2 ± 0.131 a	4.15 ± 0.040	3.79 ± 0.085	1.82 ± 0.031 b	1.96 ± 0.015 b
		MiKS 4 (Kl_2% + Si_8%)	11.9 ± 0.083 a	3.90 ± 0.150	3.79 ± 0.095	1.70 ± 0.062 b	1.95 ± 0.040 b
		*P* value	<0.001	0.086	0.489	<0.001	0.039

E-L35, veraison; E-L38, maturation; Kl, kaolin; Si, silicon; MiKS, combined formulation with Kl and Si.

At E-L35 in 2023, both untreated plants and those treated with MiKS 3 (highest Si concentration) showed similar phenolic levels, which were significantly higher than those observed in plants treated with MiKS 1 and MiKS 2.

For flavonoids, the pattern was similar, with MiKS 3-treated plants exhibiting the highest levels, with increases of 22.8%, 98.3%, and 83.5% compared to the control, MiKS 1, and MiKS 2, respectively. Additionally, MiKS 1 and MiKS 2 treated plants also exhibited significantly lower flavonoid content than control plants, with decreases of 38.1 and 33.1%, respectively. In terms of anthocyanins, vines treated with MiKS 2 and MiKS 3 showed significantly lower levels compared to the control group, with reductions of 12.6% and 10.1%, respectively, and also lower than those recorded in MiKS 1 (16.2 and 13.9% decrease, respectively, for MiKS 2 and MiKS 3).

Regarding tannins, MiKS 2-treated plants exhibited the lowest levels, significantly different from all other treatments. MiKS 1 and control plants presented intermediate values, while MiKS 3-treated plants showed the highest tannin content, with significant increases of 23.0%, 62.8%, and 101.0% compared to control, MiKS 1, and MiKS 2, respectively.

At E-L38 in 2023, significant differences in phenolic content were noted between MiKS 1 and MiKS 3, with MiKS 3 being higher, exhibiting a 16.7% increase, as observed in E-L35. For flavonoids, all treatments had smaller contents than the control but differed significantly: MiKS 1 showed the lowest content, followed by MiKS 2, and MiKS 3. Anthocyanin showed an opposite trend, being highest in MiKS 1-treated vines, significantly surpassing all other treatments, while MiKS 3-treated vines presented the lowest levels, with a reduction of 41.9% compared with MiKS 1. An increase in anthocyanin content from veraison to maturation was observed in both years and across all treatments, except for MiKS 3, where values remained similar. This increase was more pronounced in 2024, whereas in 2023 the absence of rainfall in July and August, combined with very high temperatures, likely intensified stress conditions and may have contributed to anthocyanin degradation. In contrast, for tannins, all MiKS formulations resulted in significantly lower contents compared to the control.

In 2024, at E-L35, control plants displayed the highest phenols levels, without statistical differences to MiKS3. Both MiKS 2 and MiKS 4 (the new treatment added in 2024 with 8%Si) had lower phenol contents, with 6.1% and 3.5%, decrease, respectively.

For flavonoids, all treated plants had significantly lower levels than control, with decreases of 17.7, 16.7, and 17.7%, for MiKS 2, MiKS 3, and MiKS 4, respectively. A different pattern was observed for anthocyanins and tannins. In fact, all treated plants exhibited significantly higher levels of anthocyanins when compared to the control, with increases of 91.5% (MiKS 2), 259.7% (MiKS 3), and 188.7% (MiKS 4). Regarding tannins, untreated plants also exhibited the lowest content, being significantly different from treated plants, which showed increases ranging from 35.0% to 48.8%.

At E-L38 in 2024, all treatments with Si and Kl generally resulted in significant increases in secondary metabolites compared to control plants. Indeed, treated plants exhibited significantly higher phenolic content than the control, with increases of 18.0%, 31.0%, and 30.0% for MiKS 2, MiKS 3, and MiKS 4, respectively.

Flavonoid content also followed this trend, with treated plants outperforming the control and recording increases of 35.1% for MiKS 2, 24.6% for MiKS 3, and 25.4% for MiKS 4.

In the anthocyanin quantification, MiKS 3 and MiKS 4 treatments resulted in significantly higher contents than the control, with increases of 28.1% and 53.8%, respectively. Finally, regarding tannins, untreated plants again presented the lowest content, while treated plants exhibited increases ranging between 39.0% and 61.2%.

As shown in **Figure 1**, the 2023 growing season was marked by extremely low precipitation and persistently high temperatures, during the summer months. These harsh environmental conditions likely intensified plant stress responses and altered metabolic allocation. Under such circumstances, higher Si concentrations (MiKS 2 and MiKS 3) were associated with reduced anthocyanin levels and, in the case of MiKS 2, lower tannin content. This may be explained by a reduction in oxidative signaling, which is required to activate the phenylpropanoid pathway (Chaves et al., 2009; Liang et al., 2019). Conversely, in 2024, when rainfall was more regular, especially during key phenological stages, the trend reversed. Treated vines generally exhibited significant increases in anthocyanins, flavonoids, and tannins, compounds that are crucial for taste, mouthfeel and wine evolution (Guerra, 2002; Pozzan et al., 2012; Garrido and Borges, 2013). Thus, in 2024, the MiKS 4 treatment resulted in grapes with a higher phenolic compound content, despite having a lower probable alcohol concentration. This suggests that under less extreme conditions, the treatments acted as elicitors of secondary metabolism, a role previously attributed to Si-based products (Erb and Kliebenstein, 2020; Hernandez-Apaolaza, 2022). Improvements in the plant antioxidant system following Si application under abiotic stress have been widely reported by several authors (Liang et al., 2005; Kim et al., 2017; Wang et al., 2017; Abd_Allah et al., 2019; Lukacova et al., 2019; Ahanger et al., 2020; Dinis et al., 2024a). In particular, Losada et al. (2022) demonstrated that the application of monosilicic acid in grapevines led to a higher content of total phenols, total anthocyanins, and total tannins. Similarly, foliar application of Kl has been shown to increase total phenols and anthocyanins in resulting wines (Conde et al., 2016; Dinis et al., 2016a, 2024b). The consistent increase in total phenols across all treatments at E-L38 in 2024 supports the hypothesis that Si and Kl may stimulate the activity of key enzymes such as phenylalanine ammonia-lyase (PAL), promoting phenolic biosynthesis even in the absence of acute stress conditions (Savant et al., 1996). In the present work, flavonoid dynamics showed a strong dependence on both phenological stage and year, highlighting the modulating effect of environmental context on biosynthetic pathways. In 2024, an opposite trend was observed between E-L35 and E-L38: while control plants presented the highest flavonoid content at E-L35, all treated plants surpassed the control at E-L38. This shift reinforces the idea that Si and Kl treatments interact with plant development in a context-specific manner, either by alleviating stress or by actively enhancing metabolic functions depending on the prevailing environmental conditions.

### Cuticular wax composition was influenced by Kl + Si application

**Figure 3:**
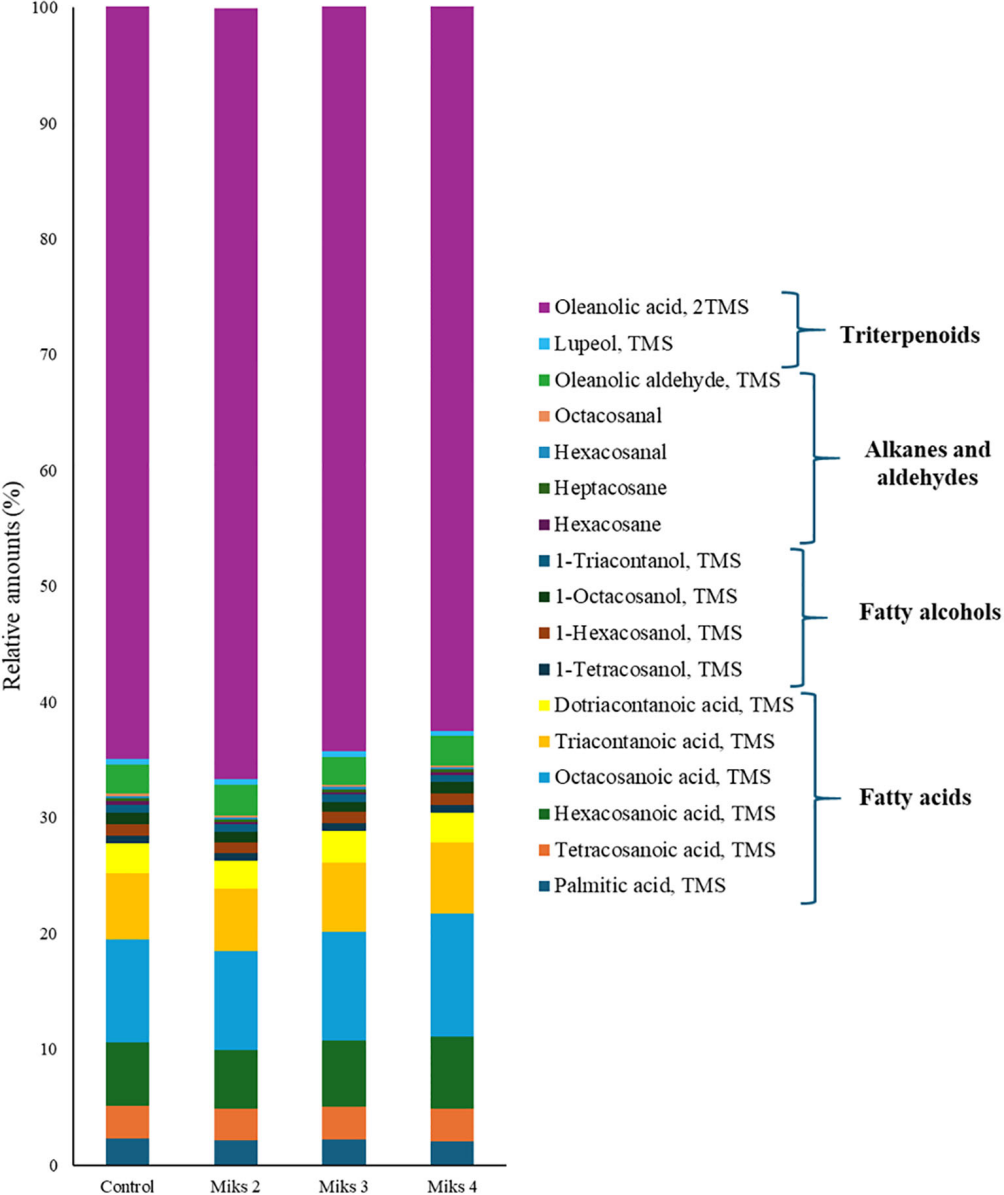
Relative amounts (%) of the most abundant aliphatic compounds in the total cuticular waxes of berries from vines with different foliar applications: untreated plants (Control), plants treated with Kl 2% and Si 4% (MiKS 2), plants treated with Kl 2% and Si 6% (MiKS 3), and plants treated with Kl 2% and Si 8% (MiKS 4) (N=25).

The outermost layer of plant organs, the cuticle, composed of epi- and intracuticular waxes, acts as a barrier against abiotic stresses such as dehydration, extreme heat or cold, and broader climate-driven alterations in temperature and precipitation patterns (Klavins and Klavins, 2020). These waxes contain very long chain (VLC, C>20) aliphatic compounds, including fatty acids, aldehydes, alcohols, ketones, alkanes, and esters (Dimopoulos et al., 2020; Zhang et al., 2021), while they may also contain triterpenoids, tocopherols, sterols, and flavonoids (Jetter and Schäffer, 2001; Buschhaus and Jetter, 2011; Bernard and Joubès, 2013; Dimopoulos et al., 2020). The chemical composition of these waxes shapes the cuticle’s microstructure and affects the adhesion and retention of water, pesticides, and airborne particles (Belding et al., 2000). **Figure 3** presents the relative abundance of the main aliphatic compounds in the total cuticular waxes of grape berries subjected to different Si and Kl treatments. As in many fleshy fruits, the grape berry cuticle is composed primarily of aliphatic waxes and triterpenoids (Lara et al., 2014). However, the results reveal clear treatment-dependent shifts in both the concentration and relative abundance of the main aliphatic compounds in grape berry cuticular waxes. Triterpenoids, derived from squalene (Klavins and Klavins, 2020), dominated the wax composition in this study, with relative abundances ranging from 63% to 67%. This is consistent with previous reports, showing that triterpenoids are the predominant compounds within the intracuticular wax layer (Jetter and Schäffer, 2001; Buschhaus and Jetter, 2011). Similarly, Zhang et al. (2021) also found that triterpenoids and fatty acids were the major components of grape berry cuticular waxes. Given the substantial amounts of triterpenoids observed in this work, grape berry cuticular wax could represent valuable bioactive compounds with potential functional properties (Zhang et al., 2021). In this study, fatty acids accounted for 26% to 30% of the total wax fraction, depending on the treatment. In contrast, fatty alcohols and alkanes/aldehydes were consistently low, each representing approximately 3% across all treatments. These low amounts were corroborated by other studies in grape berries (Zhang et al., 2021), blueberry (Chu et al., 2017) and apple (Belding et al., 1998).

**Figure 4:**
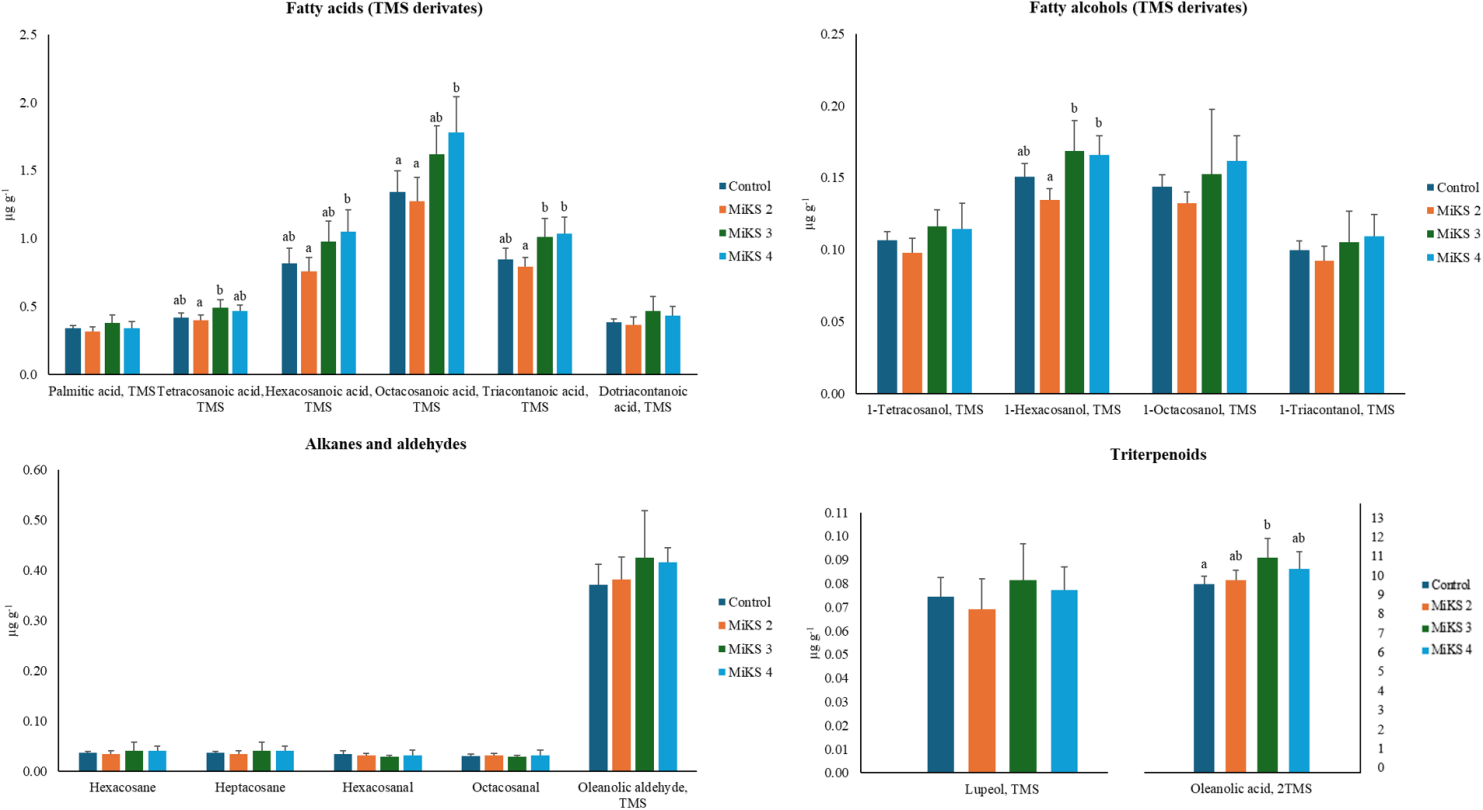
Concentration (µg g-1 FW) of the most abundant aliphatic compounds in the total cuticular waxes of berries from vines with different foliar applications: untreated plants (Control), plants treated with Kl 2% and Si 4% (MiKS 2), plants treated with Kl 2% and Si 6% (MiKS 3), and plants treated with Kl 2% and Si 8% (MiKS 4) (N=25). Results are expressed as mean ± standard deviation. Different letters indicate significant differences (P < 0.05) and the absence of letters indicates no statistically significant differences (P > 0.05) between treatments, according to Tukey’s test.

When analysing the concentration of individual compounds, significant differences among treatments were found for the fatty acids tetracosanoic, hexacosanoic, octacosanoic, and triacontanoic (**Figure 4a**). For tetracosanoic acid, MiKS 3-treated plants showed levels 23.1% higher than those treated with MiKS 2. Hexacosanoic and octacosanoic acids followed a similar trend: MiKS 2-treated plants recorded the lowest, while MiKS 4-treated plants had the highest concentrations, corresponding to increases of 38.7% and 39.5%, for hexanoic and octanoic acids, respectively. Additionally, both MiKS 3 and MiKS 4 significantly increased triacontanoic acid content compared to MiKS 2, with gains of 27.2% and 30.3%. Taken together, these results indicate that the combined application of Si and Kl promoted an accumulation of specific long-chain fatty acids, which, according to previous studies, suggests a stimulation of fatty acid elongation pathways (Samuels et al., 2008). This compositional shift may reinforce the cuticle’s barrier function by enhancing its hydrophobicity and mechanical strength, contributing to improved protection against dehydration and pathogen penetration (Yeats and Rose, 2013). Furthermore, as these long-chain fatty acids can act as signaling molecules in plant–pathogen interactions, their modulation by Si + Kl treatments points to a potential dual role in enhancing defense responses while maintaining cuticle integrity (Hegde et al., 1997; Ahmed et al., 2014).

Regarding fatty alcohols (**Figure 4b**), significant differences were observed only for 1-hexacosanol, with MiKS 3- and MiKS 4-treated plants exhibiting concentrations significantly higher than those of MiKS 2-treated plants, corresponding to increases of 26.1% and 23.9%, respectively. This selective increase may be associated with the stimulation of fatty acid reductase activity under Si influence, which can promote the conversion of long-chain fatty acids into primary alcohols (Wang et al., 2021a). Such modifications in specific wax fractions could contribute to changes in cuticle permeability and surface properties, ultimately affecting the plant’s interaction with water and pathogens.

No significant differences were found for the alkane and aldehyde fractions (**Figure 4c**), suggesting that these compound classes are less responsive to Si and Kl treatments under the conditions tested. Interestingly, alkanes are generally regarded as key contributors to minimizing cuticular water loss by increasing the hydrophobicity of the cuticle surface (Klavins and Klavins, 2020), which implies that maintaining stable levels of these compounds may help preserve baseline water retention even when other wax fractions are modulated. Conversely, significant differences were found for oleanolic acid (**Figure 4d**), with MiKS 3-treated plants showing concentrations 14.2% higher than the control. This triterpenoid was consistently the most abundant across all treatments, which aligns with previous reports that oleanolic acid and its precursors are the main triterpenoids in grapes (Radler, 1965). Notably, the proportion of cuticular triterpenoids and aliphatic waxes can vary greatly among grapevine cultivars; for example, oleanolic acid may account for up to 42% of the total wax content in Muscat d’Alsace berries, whereas it is completely absent in Sylvaner berries (Pensec et al., 2014). The significant increase observed in MiKS 3-treated plants reinforces the idea that Si can promote triterpenoid biosynthesis, enhancing the accumulation of compounds that play key roles in pathogen defense and berry firmness (Szakiel et al., 2012; Jetter and Schäffer, 2001; Klavins and Klavins, 2020). These findings are in line with studies indicating that Si can stimulate cuticle deposition and alter its chemical composition, potentially improving water retention and strengthening resistance to pathogens (Guntzer et al., 2012; Wang et al., 2021a). Indeed, higher cuticular content has been reported to correlate negatively with water loss, thereby extending fruit shelf life (Lara et al., 2014). On the other hand, since Kl does not penetrate plant tissues or modulate cuticle or wax biosynthetic pathways, its effect is predominantly physical and reflective. The white particle film forms a protective barrier on the surface of the epidermis and cuticle, reducing absorbed solar radiation, berry temperature, and water loss (Glenn et al., 2010). This barrier can also help limit wax degradation by preventing fusion or structural rearrangement under excessive heat (Dimopoulos et al., 2020); however, it does not stimulate the synthesis of new lipid fractions.

MiKS treatments enhanced long-chain fatty acids, 1-hexacosanol, and oleanolic acid, reinforcing cuticle hydrophobicity and defense, thereby improving berry protection under stress.

### The influence of treatments on the berry histological parameters

**Figure 5:**
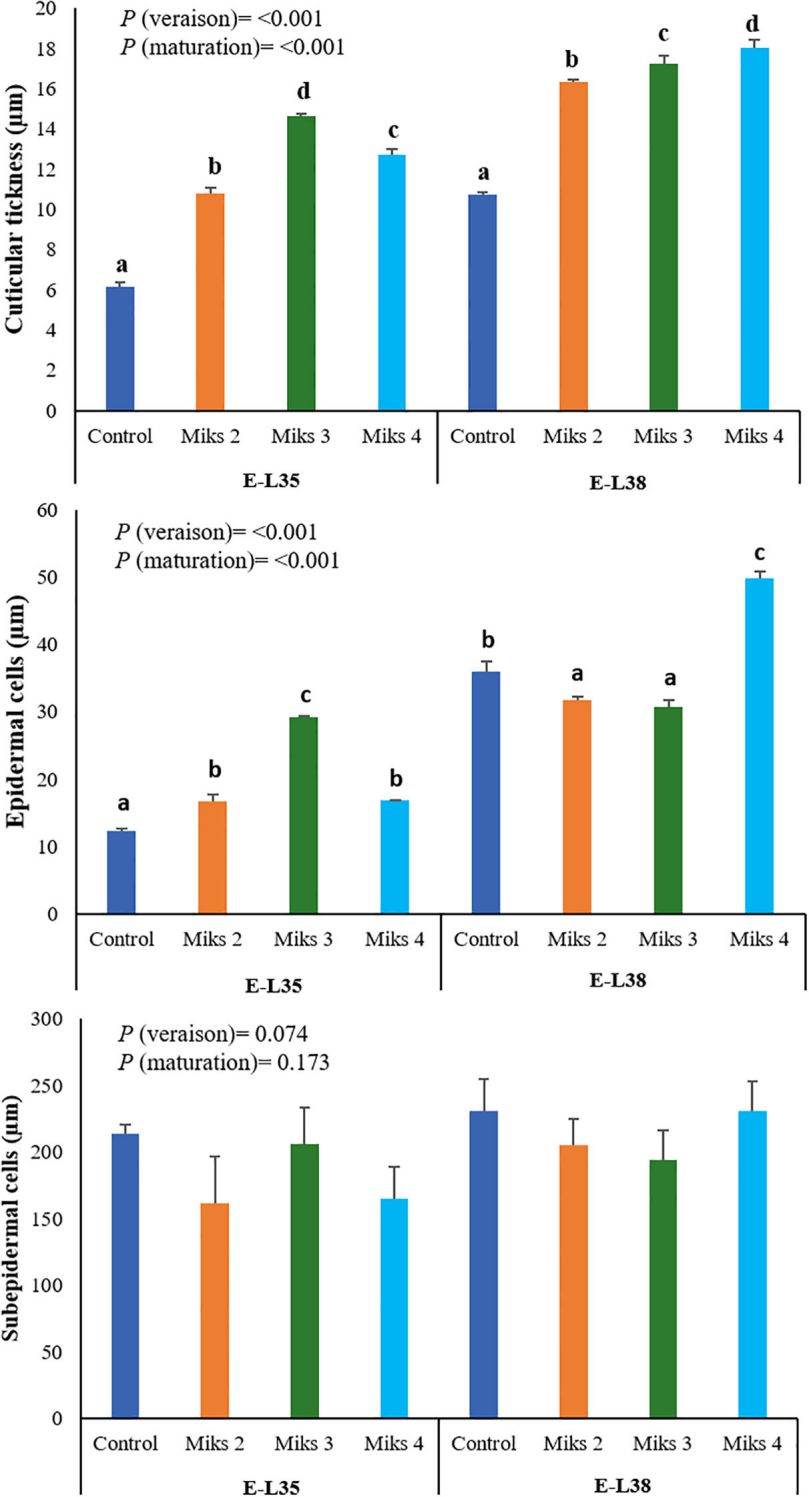
Histological parameters (cuticular thickness, and epidermal and subepidermal cells) of berries from vines with different foliar applications: untreated plants (Control), plants treated with Kl 2% and Si 2% (MiKS 1), plants treated with Kl 2% and Si 4% (MiKS 2), plants treated with Kl 2% and Si 6% (MiKS 3), and plants treated with Kl 2% and Si 8% (MiKS 4) (N=6). Results are expressed as mean ± standard deviation. Different letters indicate significant differences (*P* < 0.05) and the absence of letters indicates no statistically significant differences (*P* > 0.05) between treatments, within the same phenological stage, according to Tukey’s test.

The histological parameters assessed in 2024 (**Figure 5**) provide further evidence of how Si and Kl applications can modulate the structural features of grape berry skins. During E-L35, all treated plants showed greater cuticle thickness and larger epidermal cells compared to the control, with MiKS 3-treated fruits displaying the highest values for both traits. Increased cuticle thickness is generally associated with reduced transpirational water loss and improved tolerance to dehydration, while also serving as a physical barrier that can hinder pathogen penetration. The concomitant enlargement of epidermal cells may further contribute to structural reinforcement of the fruit surface under stress conditions. At E-L38, the same trend was observed for cuticle thickness: MiKS 2-, MiKS 3-, and MiKS 4-treated berries exhibited significantly thicker cuticles than the control, with increases of 52.7%, 61.2%, and 68.5%, respectively. Interestingly, while cuticle thickness increased consistently with treatment, epidermal cell size was highest in untreated plants and in those treated with MiKS 4. This may indicate that cuticular deposition and epidermal expansion are not strictly correlated, and that MiKS 4 might promote structural reinforcement of the cuticle without limiting epidermal cell growth, possibly through altered metabolic allocation or signaling pathways associated with stress responses. The thickness of the subepidermal cell layer remained unchanged across treatments, indicating that Si and Kl primarily affect the outermost protective layers. Indeed, Si is well known for strengthening cell walls and stimulating the biosynthesis of cutin and waxes, which together contribute to thicker, more robust cuticles that reduce water loss and enhance resistance to abiotic and biotic stressors (Guntzer et al., 2012; Zargar et al., 2019; Wang et al., 2021b; Zhu and Li, 2021). In contrast, Kl acts as a reflective particle film that lowers fruit surface temperatures and reduces sunburn, indirectly supporting epidermal integrity and cuticle development under heat stress (Glenn and Puterka, 2010).

### Kl+Si mixtures application regulates fruit physiological behavior

**Figure 6:**
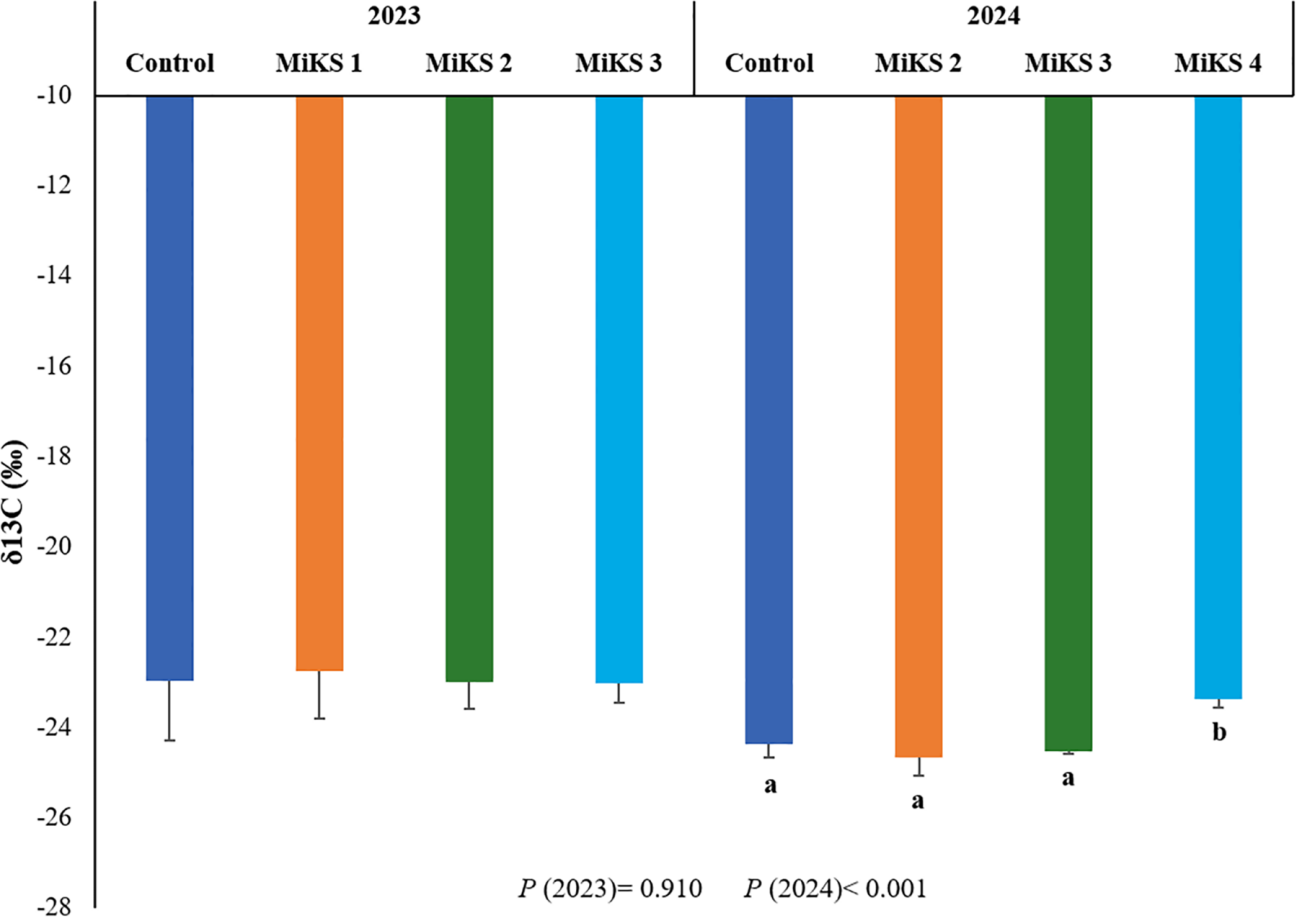
δ^13^C composition of berries from vines with different foliar applications: untreated plants (Control), plants treated with Kl 2% and Si 2% (MiKS 1), plants treated with Kl 2% and Si 4% (MiKS 2), plants treated with Kl 2% and Si 6% (MiKS 3), and plants treated with Kl 2% and Si 8% (MiKS 4) (N=9). Results are expressed as mean ± standard deviation. Different letters indicate significant differences (*P* < 0.05) and the absence of letters indicates no statistically significant differences (*P* > 0.05) between treatments, within the same year, according to Tukey’s test.

The δ¹³C isotopic signature is widely recognized as an integrative indicator of intrinsic water-use efficiency (WUE) in plants, since higher δ¹³C values typically reflect lower stomatal conductance and greater CO_2_ assimilation efficiency under limited water availability (Cernusak et al., 2013). The quantified results of the δ¹³C isotopic composition under different treatments at E-L38 in both 2023 and 2024 are presented in **Figure 6**. Significant differences were observed only in 2024, when berries from MiKS 4-treated plants exhibited significantly higher δ¹³C values, with increases of 4.27%, 5.55%, and 4.70% compared to the control, MiKS 2, and MiKS 3-treated plants, respectively. The enrichment in ^13^C, which results in higher δ¹³C values, for MiKS 4-treated vines suggests that this formulation may have promoted physiological adjustments leading to improved WUE during the 2024 growing season, which experienced milder drought conditions than 2023. Notably, this trend is consistent with findings from our research group, which also reported increased WUE in MiKS 4-treated plants at E-L38 in 2024 (Pereira et al., 2025). These results align with the enhanced cuticular wax deposition, increased cuticle thickness, and improved epidermal cell density and organization observed in the present work, for MiKS 4-treated plants, all of which likely contributed to reduced transpirational water loss and tighter stomatal regulation. In the work developed by Zhang et al. (2020), a positive correlation was observed between Si content and foliar δ^13^C. Moreover, foliar application of Si, especially at higher doses, appears to reinforce structural barriers that limit water loss in the fruit under moderate stress (Guntzer et al., 2012; Wang et al., 2021a). These results suggest that the observed differences in δ¹³C are mainly attributable to Si, since foliar Kl applications alone usually do not change carbon isotope composition significantly. Taken together, the δ¹³C enrichment observed in 2024 and the related anatomical changes in berry tissues highlight the multiple ways these treatments strengthen fruit robustness under drought conditions. By supporting both physiological processes and structural defenses, they help maintain berry water status and quality under variable water regimes (Cernusak et al., 2013; Krishankumar et al., 2025). Conversely, the lack of significant differences observed in 2023 suggests that the applied formulations were particularly effective in mitigating the effects of the extremely warm conditions that year.

### Impacts of the different formulations on the hormonal signaling

**Figure 7:**
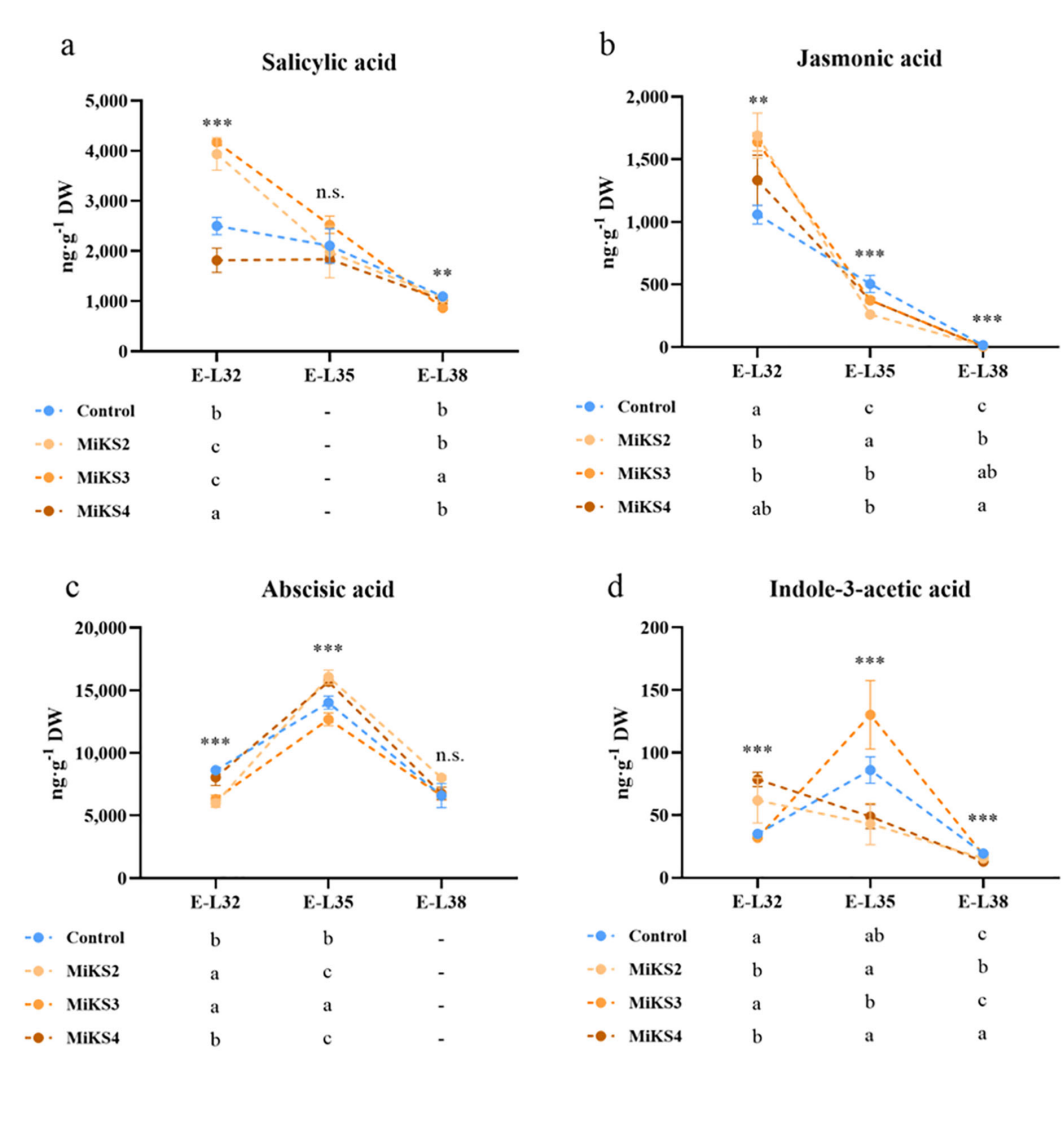
Hormonal profile (salicylic acid, jasmonic acid, abscisic acid, and indol-3-acetic acid) of berries from vines with different foliar applications: untreated plants (Control), plants treated with Kl 2% and Si 2% (MiKS 1), plants treated with Kl 2% and Si 4% (MiKS 2), plants treated with Kl 2% and Si 6% (MiKS 3), and plants treated with Kl 2% and Si 8% (MiKS 4) (N=9). Results are expressed as mean ± standard deviation. Different letters indicate significant differences (*P* < 0.05) and the absence of letters indicates no statistically significant differences (*P* > 0.05) between treatments, within the same phenological stage, according to Tukey’s test.

**Figure 7** shows the phytohormonal profile of grape berries from vines treated with different Kl and Si formulations, revealing a dynamic interaction between treatments and hormonal regulation. Statistically significant differences among treatments were observed across the three phenological stages, except for salicylic acid (SA) at E-L35 and abscisic acid (ABA) at E-L38. At E-L32, the highest SA content was found in MiKS 3-treated plants, with an increase of 67.0% compared to the control and 130.0% relative to MiKS 4. MiKS 2-treated plants also presented significantly higher SA levels than control and MiKS 4 plants. This suggests an early activation of systemic acquired resistance pathways in plants treated with MiKS 2 and 3, which can prime the synthesis of phenolics and flavonoids (Pieterse et al., 2009; Erb and Kliebenstein, 2020). For JA, the highest levels were observed in MiKS 2-treated plants, followed by MiKS 3, with both showing significantly higher values than the control (59.6% and 54.7% increase, respectively). Elevated JA levels further indicate enhanced defense signaling, in line with reports that JA plays a key role in biotic stress responses (Pieterse et al., 2009; Dinis et al., 2018a). In contrast, ABA content was significantly lower in MiKS 2 and MiKS 3-treated plants (–4.4% and –36.8% compared to control), pointing to a possible downregulation of drought stress signaling, consistent with observations that ABA mediates stomatal closure and water deficit responses (Chaves et al., 2009). Meanwhile, IAA levels were highest in MiKS 4-treated plants, with increases of 146.2% and 123.6%, compared with MiKS3 and control plants, respectively, which may reflect enhanced cell expansion and berry growth in these plants, as the auxins are central to berry development and ripening (Böttcher et al., 2010).

At E-L35, although MiKS 3-treated plants showed the highest SA content, differences were not statistically significant. On the other hand, JA levels were highest in the control plants and decreased significantly in MiKS 2 (–93.4%), MiKS 3 (–34.7%), and MiKS 4 (–34.5%) treatments, indicating that Si and Kl may reduce stress perception or redirect resources towards growth, aligning with findings that Kl films mitigate heat stress and promote physiological stability (Glenn and Puterka, 2010). Similarly to E-L32, ABA content remained lowest in MiKS 3-treated plants, with control, MiKS 2 and MiKS 4 being 9.5%, 21.0% and 19.1% higher, respectively, again suggesting a dampened drought-response signal in these plants (Chaves et al., 2009). In contrast, IAA concentrations peaked in MiKS 3-treated plants (+202.7% vs MiKS 2, + 166.5% vs MiKS 4), reinforcing the idea that MiKS 3 formulation can stimulate auxin-mediated growth during berry ripening (Böttcher et al., 2010).

At E-L38, MiKS 3-treated plants showed the lowest SA content (–20.7% vs control), which may indicate a return to basal defense levels as ripening finalizes. For JA, all MiKS treatments led to significantly lower levels than the control, with reductions of 59.5% (MiKS 2), 124.5% (MiKS 3), and 224.5% (MiKS 4). The reduced JA levels observed in treated plants at harvest could reflect lower stress pressure or a shift towards ripening-associated processes (Erb and Kliebenstein, 2020). ABA differences were no longer significant at this stage. For IAA, the highest content was found in the control and MiKS 3 treatments. At the same time, MiKS 2 and MiKS 4 showed decreases of 25.4% and 50.7%, respectively, relative to the control. The reduction in IAA levels may suggest that certain formulations limit excessive berry enlargement, as lower auxin activity is known to restrict cell expansion and thereby reduce berry growth (Böttcher et al., 2010). These results align with recent findings in other crops showing that foliar Si applications can modulate phytohormonal pathways in fruit tissues. Specifically, the reductions in ABA levels and increases in SA and IAA under certain Si-based formulations observed in this work are consistent with Yang et al. (2024), who reported that appropriate doses of foliar Si significantly decreased ABA while enhancing gibberellin, auxin, and salicylic acid levels in tomato fruits. Previous studies also reported that Kl applications, beyond their physical effect, can also modulate hormonal balance in grape berries by reducing ABA and adjusting SA and IAA levels, helping to fine-tune drought responses and ripening (Frioni et al., 2020; Bernardo et al., 2022).

## Conclusion

In general, this work provides evidence that the combined foliar application of Kl and Si represents a promising and practical strategy to mitigate the negative impacts of drought and heat stress on grape berry development, composition, and quality. The synergistic effects observed here, especially in 2024, demonstrate that these treatments modulate secondary metabolite accumulation, enhance the chemical composition and structure of cuticular waxes, and promote beneficial histological adaptations that strengthen the berry’s physical barriers, reducing transpirational water loss and improving tolerance to dehydration. Furthermore, the increase in δ¹³C values and the fine-tuning of key phytohormonal pathways, notably the reductions in ABA and the adjustments in SA, JA, and IAA, highlight the ability of these foliar sprays to influence plant stress responses. This integrated action supports improved water-use efficiency, reduced probable alcohol, increased tartaric acid content with no lasting effect over total acidity, and overall fruit quality under increasingly variable climatic conditions. Nonetheless, some of this variation could reflect slight differences in berry maturity, which were not fully accounted for in this work. Together, these findings reinforce the potential of combining Kl and Si as part of sustainable viticulture management practices to preserve fruit quality, maintain yield stability, and enhance vineyard adaptability in regions most exposed to the challenges of climate change. Considering all the parameters evaluated, MiKS 3 emerged as the most promising formulation, as it promoted significant improvements in plant performance while showing only minor differences when compared to MiKS 4. Therefore, the increased cost associated with the use of 8% Si in MiKS 4 may not be justified, given the similar outcomes achieved with the 6% Si concentration in MiKS 3.

## Data Availability

The raw data supporting the conclusions of this article will be made available by the authors, without undue reservation.
